# Effects of TiO_2_ Nanotubes and Reduced Graphene Oxide on *Streptococcus mutans* and Preosteoblastic Cells at an Early Stage

**DOI:** 10.3390/ijms25021351

**Published:** 2024-01-22

**Authors:** Min-Kyung Ji, Hyeonji Kim, Geonwoo Jeong, Won-Jae Kim, Je-Hwang Ryu, Hoonsung Cho, Hyun-Pil Lim

**Affiliations:** 1 Dental 4D Research Center, Chonnam National University, 33 Yongbong-ro, Buk-gu, Gwangju 61186, Republic of Korea; perizimin@gmail.com; 2 Department of Prosthodontics, School of Dentistry, Chonnam National University, 33 Yongbong-ro, Buk-gu, Gwangju 61186, Republic of Korea; otwohh@naver.com; 3 Department of Materials Science & Engineering, Chonnam National University, 77 Yongbong-ro, Buk-gu, Gwangju 61186, Republic of Korea; rjsdn7927@daum.net; 4 Department of Oral Physiology, School of Dentistry, Chonnam National University, 33 Yongbong-ro, Buk-gu, Gwangju 61186, Republic of Korea; wjkim@jnu.ac.kr; 5 Department of Pharmacology and Dental Therapeutics, School of Dentistry, Chonnam National University, 77 Yongbong-ro, Buk-gu, Gwangju 61186, Republic of Korea; jesryu@jnu.ac.kr

**Keywords:** TiO_2_ nanotubes, reduced graphene oxide, nonthermal atmospheric plasma, *Streptococcus mutans*, preosteoblastic cells

## Abstract

The effects of TiO_2_ nanotube (TNT) and reduced graphene oxide (rGO) deposition onto titanium, which is widely used in dental implants, on *Streptococcus mutans* (*S. mutans*) and preosteoblastic cells were evaluated. TNTs were formed through anodic oxidation on pure titanium, and rGO was deposited using an atmospheric plasma generator. The specimens used were divided into a control group of titanium specimens and three experimental groups: Group N (specimens with TNT formation), Group G (rGO-deposited specimens), and Group NG (specimens under rGO deposition after TNT formation). Adhesion of *S. mutans* to the surface was assessed after 24 h of culture using a crystal violet assay, while adhesion and proliferation of MC3T3-E1 cells, a mouse preosteoblastic cell line, were evaluated after 24 and 72 h through a water-soluble tetrazolium salt assay. TNT formation and rGO deposition on titanium decreased *S. mutans* adhesion (*p* < 0.05) and increased MC3T3-E1 cell adhesion and proliferation (*p* < 0.0083). In Group NG, *S. mutans* adhesion was the lowest (*p* < 0.05), while MC3T3-E1 cell proliferation was the highest (*p* < 0.0083). In this study, TNT formation and rGO deposition on a pure titanium surface inhibited the adhesion of *S. mutans* at an early stage and increased the initial adhesion and proliferation of preosteoblastic cells.

## 1. Introduction

The loss of supporting tissues around dental implants caused by bacterial infection is one of the many causes of implant failure [1,2]. Simonis et al. [3] reported in a follow-up study over 10 to 16 years that, despite the implant success rate of 82.94%, the biological failure rate of the implants was 16.94% and the technical failure rate was 31.09. Peri-implantitis is a term used to describe cases in which inflammation generated in the tissues surrounding osseointegrated implants has destructively progressed to the extent that it affects implant stability [4]. The bacterial colonization pattern in the biofilm of natural teeth is similar to that of dental implants with peri-implantitis [5].

*Streptococcus mutans* (*S. mutans*) is a bacterium that easily attaches to the tooth surface and tissue, making large contributions to initial biofilm formation [6]. *S. mutans* induces the adhesion and proliferation of bacteria that otherwise lack adhesion mechanisms, causing periodontal disease and increasing the likelihood of recurrence after treatment [7]. The initial biofilm formed by *S. mutans* facilitates late bacterial colonization through interactions with other bacteria, including *Aggregatibacter actinomycetemcomitans* (*Aa*), *Fusobacterium nucleatum* (*Fn*), and *Porphyromonas gingivalis* (*P. gingivalis*), and this biofilm accumulates down to the subgingival area [8]. Therefore, to ensure successful implantation, it is important to promote the osseointegration of implant surfaces by suppressing the adhesion of bacteria, such as *S. mutans*, onto the implant surfaces after implantation. It has been reported that the topography of implant surfaces affects not only the adhesion of cells but also that of bacteria [9,10]. Various attempts at surface modification to increase the degree of bonding between implant surfaces and bone tissues have been made [11]. On titanium surfaces exposed to air, oxide films such as TiO, amorphous TiO_2_, TiO_2_ (anatase), TiO_2_ (brookite), TiO_2_ (rutile), Ti_2_O_3_, and Ti_x_O_y_ are formed [12]. The titanium oxide film promotes osseointegration formation and corrosion resistance, but the naturally occurring oxide film is not dense, and the film layer is thin. Anodic oxidation, one of the methods for modifying the titanium surface, is a method of electrochemically forming a thin, rough, porous amorphous TiO_2_ oxide film on the titanium surface [13]. The formation of amorphous TiO_2_ nanotubes (TNTs) on titanium surfaces by anodic oxidation increased the surface area of titanium, leading to enhanced cell adhesion, proliferation, and differentiation. This result has been reported to improve the bonding strength between the dental implant and the surrounding bone and promote osteogenesis [14,15,16].

Graphene oxide (GO) nanomaterials have been increasingly studied for biomedical applications including drug delivery carriers, imaging agents, biosensors, and tissue engineering scaffolds, due to their outstanding physicochemical, optical, electrical, and mechanical properties [17,18]. In particular, the potential of graphene and its derivatives as 2D culture platforms for the differentiation of various types of stem cells towards osteogenesis has attracted considerable attention [19]. GO is a material with a monolayer structure in which carbon atoms are bonded covalently in a hexagonal honeycomb shape. Since GO has various functional groups on its surface, including epoxy, hydroxyl, carbonyl, and carboxylic acid, it was reported that it could serve as a GO-based composite [20,21,22]. GO is typically synthesized by mechanically or chemically exfoliating bulk graphite and then depositing it on a metal catalyst using chemical vapor deposition (CVD) and Hummer’s technique [23,24,25]. GO is hydrophilic due to functional groups such as carboxyl, hydroxyl, and epoxy and has a high mechanical flexibility. However, these GO deposition methods have disadvantages that include the potential for contamination by residue from the solution used during manufacturing and the generation of toxic gas [26,27,28].

Plasma is a partially ionized gas containing highly reactive particles such as electrically excited atoms, molecules, and free radical species [29]. Various gases, through atmospheric plasma, break down carbon molecules into carbon atoms and create GO [30,31]. GO deposition using atmospheric plasma is simple and cost-effective and has the advantage that no other additives or by-products are produced during the production of GO [32]. In this study, reduced GO (rGO) was synthesized using the atmospheric plasma method, which allows the synthesis of rGO without using any catalyst for effective deposition on various substrates. GO becomes rGO through a reduction process, which is hydrophobic. Hydrophobic rGO was synthesized by direct deposition using a mixture of methane and argon gas while super hydrophobic [33].

Most studies on graphene have focused on whether it is toxic in vitro and in vivo [34,35]. On the other hand, the in vivo bioactive potential of graphene and related materials remains to be studied. Although graphene-based materials have shown appropriate biocompatibility when used in orthopedic implants, little research has been performed to specifically test the biocompatibility of graphene for dental applications. Lee et al. [36] reported that rGO-based composite materials fabricated to accelerate bone regeneration have the potential to stimulate osteogenesis. Williams et al. [37] reported that particle size, shape, and concentration of graphene-based materials for applications are major factors affecting cytotoxicity, antibacterial properties, and cell differentiation ability.

This study aimed to determine the biofilm formation of *S. mutans* and the activity of MC3T3-E1 cells, a mouse preosteoblastic cell line, on titanium surfaces fabricated with TNTs and rGO.

## 2. Results

### 2.1. Surface Characteristics

**Figure 1:**
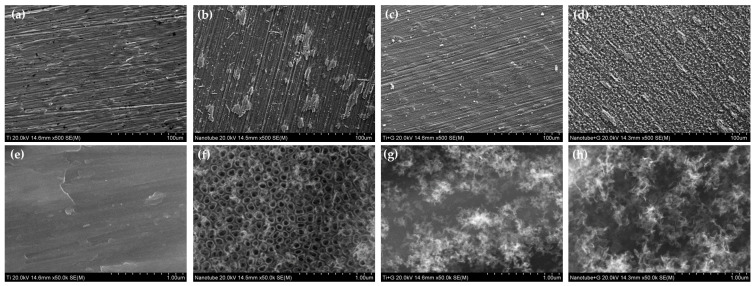
Surface morphology of (**a**,**e**) polished titanium (control group), (**b**,**f**) TiO_2_ nanotube titanium (Group N), (**c**,**g**) reduced graphene oxide-deposited titanium (Group G), and (**d,h**) reduced graphene oxide-deposited TiO_2_ nanotube titanium (Group NG) ((**a**–**d**): magnification = 500, (**e**–**h**): magnification = 50,000).

In the scanning electron microscope (SEM) observations, TNTs were formed in Group N. It was also observed that, in Group G, rGO was deposited in the form of a cloud, and that, in Group NG, TNTs that formed on the surface were covered by the deposited rGO (Figure 1).

**Figure 2:**
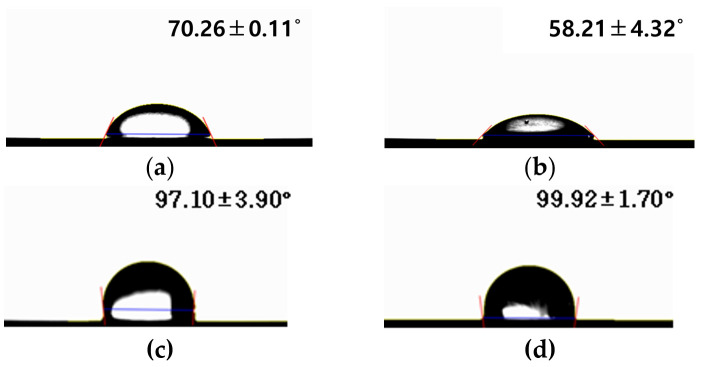
Contact angle of (**a**) polished titanium (control group), (**b**) TiO_2_ nanotube titanium (Group N), (**c**) reduced graphene oxide-deposited titanium (Group G), and (**d**) reduced graphene oxide-deposited TiO_2_ nanotube titanium (Group NG).

Surface roughness was measured to be 0.145 ± 0.036 μm for the control group, 0.204 ± 0.030 μm for Group N, 0.245 ± 0.032 μm for Group G, and 0.289 ± 0.021 μm for Group NG. The control group showed the lowest roughness, followed by Group N, Group G, and finally, Group NG, which had the highest roughness. The change in contact angle according to TNT formation and rGO deposition is shown in Figure 2. The contact angle was measured to be 70.26 ± 0.11° for the control group, 58.21 ± 4.32° for Group N, 97.10 ± 3.90° for Group G, and 99.92 ± 1.70° for Group NG. The control group showed the lowest contact angle, followed by Groups N, G, and NG.

**Figure 3:**
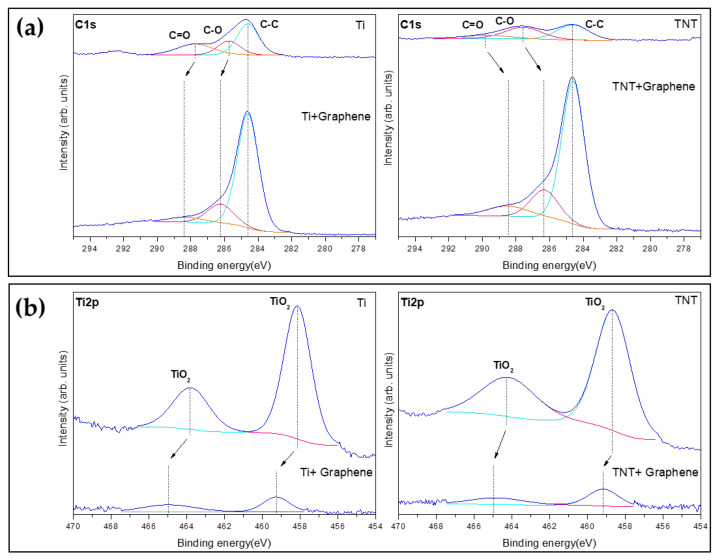
Chemical composition analysis of the reduced graphene oxide deposits. XPS high-resolution spectra at (**a**) C1s and (**b**) Ti2p. The Cls peak has decomposed into ClsA (cyan line), C1sB (magenta line), C1sC (orange line) and the Ti2p peak has decomposed into Ti2p1 (magenta line), Ti2p3 (cyan line). Dashed lines indicate that the binding energy of each peak has shifted.

The results of a chemical composition analysis of the rGO deposits are shown in Figure 3. Of the three C1 peaks (284.6, 285.7, and 287.8 eV) detected in the titanium samples, the 284.6 eV peak reflects C-C bonded carbon, while the 285.7 eV peak reflects C-O bonded carbon, and the 287.8 eV peak reflects C=O bonded carbon. In the case of Ti2p, two distinct peaks were observed at 463.8 and 458.1 eV, indicating TiO_2_.

After rGO deposition, the C1 peaks of the Ti samples increased, and the low peaks between 288.3 and 286.2 eV were slightly shifted to a higher bonding energy. The C1 peaks of TNT also increased, while the low peaks between 289.7 and 287.6 eV were slightly shifted to a lower bonding energy. The Ti2p peaks were slightly shifted to a higher bonding energy.

**Figure 4:**
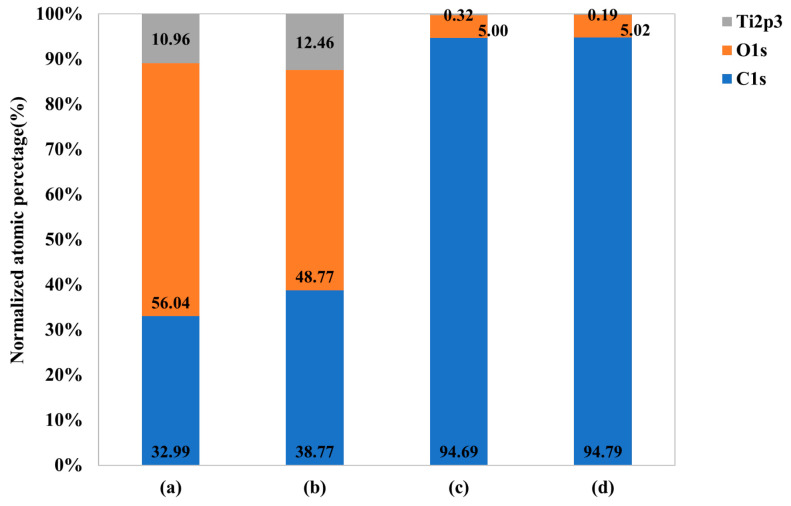
Normalized atomic percentage of each element on (**a**) polished titanium (control group), (**b**) TiO_2_ nanotube titanium (Group N), (**c**) reduced graphene oxide-deposited titanium (Group G), and (**d**) reduced graphene oxide-deposited TiO_2_ nanotube titanium (Group NG).

Based on the peaks shown in XPS survey profiles, the atomic percentage of each control and experimental group was graphed (Figure 4). For the control group, the carbon content of the surface was 32.99 at%, the oxygen content was 56.04 at%, and the titanium content was 10.96 at%. For Group N, the carbon content of the surface was 38.77 at%, the oxygen content was 48.77 at%, and the titanium content was 12.46 at%. For Group G, it was observed that the carbon content of the surface was significantly increased to 94.69 at%, the oxygen content was 5.00 at%, and the titanium content was significantly decreased to 0.32 at%. For Group NG, the carbon content of the surface was 94.79 at%, the oxygen content was 5.02 at%, and the titanium content was 0.19 at%.

**Figure 5:**
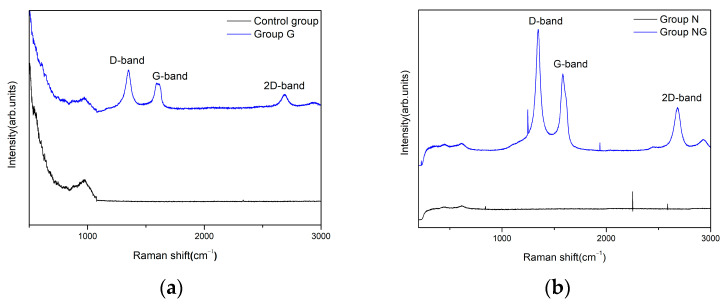
Characterization of reduced graphene oxide-deposited surfaces using Raman spectroscopy on (**a**) polished titanium (control group) and reduced graphene oxide-deposited titanium (Group G) and (**b**) TiO_2_ nanotube titanium (Group N) and reduced graphene oxide-deposited, TiO_2_ nanotube titanium (Group NG).

The samples were analyzed via Raman spectroscopy to validate the presence of rGO. The D, G, and 2D bands in Group G occurred at 1350, 1593, and 2683 cm^−1^, respectively, indicating that rGO was successfully synthesized (Figure 5a). Similarly, a major band position at 1348, 1583, and 2683 cm^−1^, corresponding to rGO, was detected in Group NG (Figure 5b). The relative intensity ratio of I_2D_/I_G_ can be used to distinguish the number of layers of graphene. The intensities of Group G, as shown in Figure 5a, are 550, 496, and 448 corresponding to the D, G, and 2D bands, respectively, and the intensity ratio for I_2D_/I_G_ was 0.9, showing a multi-layered rGO. The intensities of Group NG, shown in Figure 5b, are 810, 606, and 453 for the D, G, and 2D peaks, respectively, and its ratio for I_2D_/I_G_ is 0.74, which corresponds to a multi-layered rGO. The I_D_/I_G_ ratio of Group G and NG is 1.1 and 1.3, respectively. 

### 2.2. Assessment of the Ability to Inhibit Biofilm Formation

**Figure 6:**
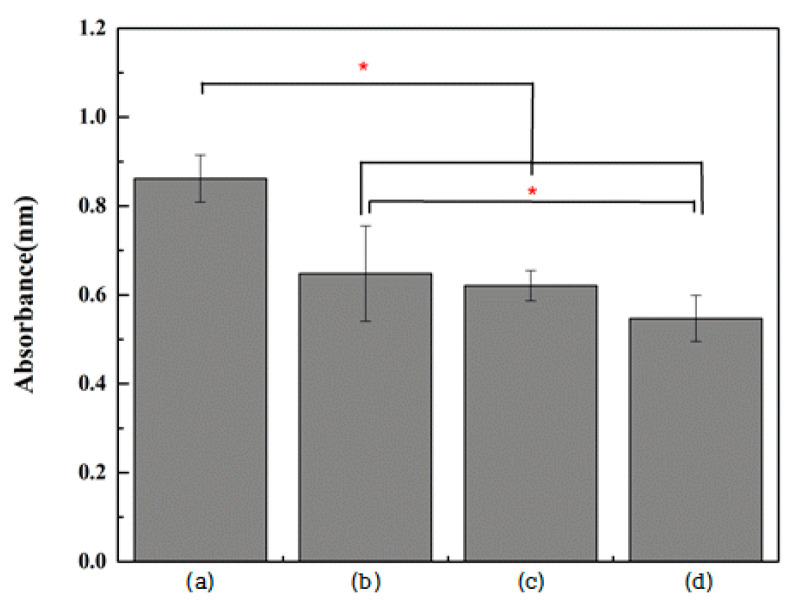
Absorbance level of *S.mutans* after incubation for 24 h on (**a**) polished titanium (control group), (**b**) TiO_2_ nanotube titanium (Group N), (**c**) reduced graphene oxide-deposited titanium (Group G), and (**d**) reduced graphene oxide-deposited TiO_2_ nanotube titanium (Group NG) (the result of one-way ANOVA test, *: significant at *p* < 0.05).

By assessing the adhesion of *S. mutans* to specimens, it was found that adhesion was significantly reduced in all the experimental groups (Groups N, G, and NG) compared with the control group; further, the adhesion of *S. mutans* in Group NG was significantly lower than in Group N (*p* < 0.05; Figure 6).

### 2.3. Assessment of Osteoblastic Activity

**Figure 7:**
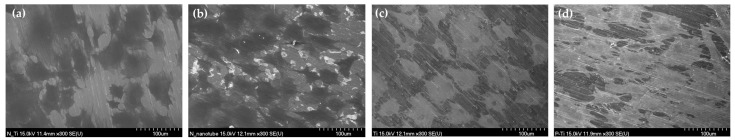
SEM images of MC3T3-E1 cells after incubation for 4 h on (**a**) polished titanium (control group), (**b**) TiO_2_ nanotube titanium (Group N), (**c**) reduced graphene oxide-deposited titanium (Group G), and (**d**) reduced graphene oxide-deposited TiO_2_ nanotube titanium (Group NG) ((**a**–**d**): magnification = 300).

In total, 4 × 10^4^ cells/mL of MC3T3-E1 cells were dispensed onto the specimen, and after culturing for 4 h, the adhesion of the cells was observed using an SEM. In all specimens, MC3T3-E1 cells were observed to be well attached. Compared to the control group, the number of cells attached to the surface was higher in Group N, G, and NG. No obvious differences were observed between the TNT-formed surface and the rGO-deposited surface (Figure 7).

**Figure 8:**
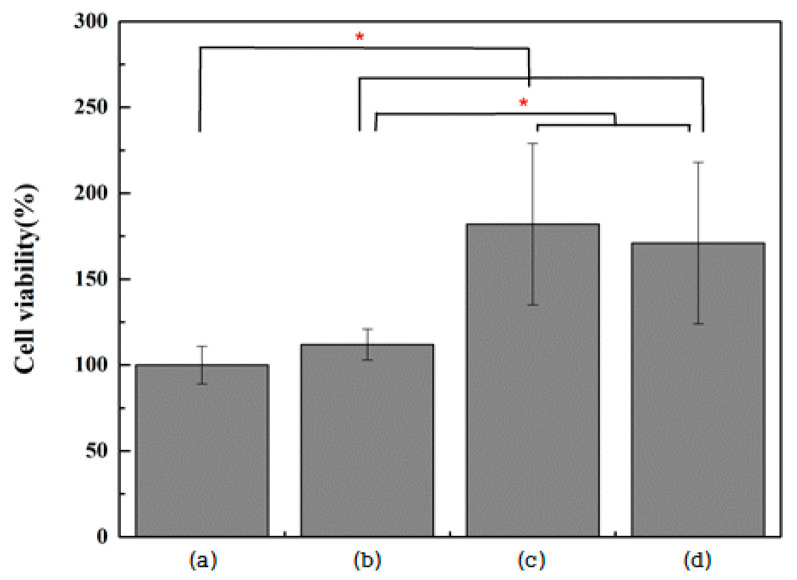
Evaluation of MC3T3-E1 cell viability after incubation for 24 h on (**a**) polished titanium (control group), (**b**) TiO_2_ nanotube titanium (Group N), (**c**) reduced graphene oxide-deposited titanium (Group G), and (**d**) reduced graphene oxide-deposited TiO_2_ nanotube titanium (Group NG) (the result of Kruskal–Wallis test, *: significant at *p* < 0.0083).

**Figure 9:**
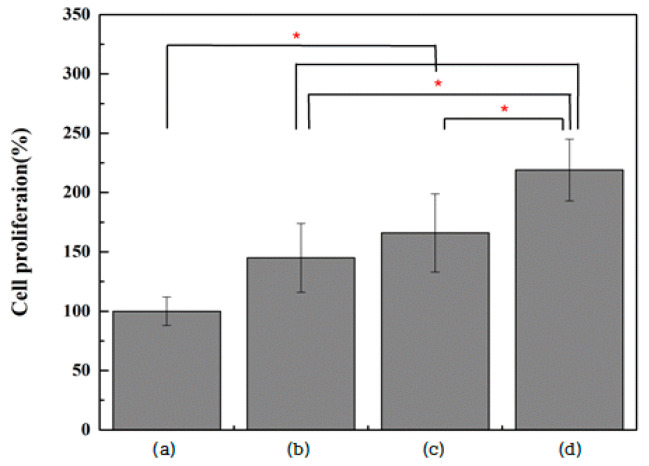
Evaluation of MC3T3-E1 cell proliferation after incubation for 72 h on (**a**) polished titanium (control group), (**b**) TiO_2_ nanotube titanium (Group N), (**c**) reduced graphene oxide-deposited titanium (Group G), and (**d**) reduced graphene oxide-deposited TiO_2_ nanotube titanium (Group NG) (the result of Kruskal–Wallis test, *: significant at *p* < 0.0083).

The viability of MC3T3-E1 cells was significantly increased in all three experimental groups compared with the control group (*p* < 0.0083). The surfaces of the two rGO-coated groups (Group G and NG) were associated with significantly higher MC3T3-E1 cell viability compared with those on which TNTs were formed (Group N) (*p* < 0.0083; Figure 8). The proliferation of MC3T3-E1 cells was significantly increased in all three experimental groups compared with the control group (*p* < 0.0083). In Group NG, cell proliferation was significantly higher than that of surfaces on which TNTs were formed (Group N) and those on which rGO was coated (Group G) (*p* < 0.0083; Figure 9).

## 3. Discussion

Bacterial adhesion and osteoblastic activity are significantly affected by the surface morphology and roughness of the implants. This study sought to examine the extent to which TNT formation and rGO deposition onto titanium surfaces inhibit the adhesion of *S. mutans* and activate osteoblasts. High surface roughness increases the accumulation of biofilm [7], as well as the initial adhesion of osteoblasts [38,39]. In the crystal violet assay conducted in this study, it was observed that as surface roughness increased—in the order of the control group (0.145 ± 0.036 μm), Group N (0.204 ± 0.030 μm), Group G (0.245 ± 0.032 μm), and Group NG (0.289 ± 0.021 μm)—the adhesion of *S. mutans* decreased. This result contradicts the findings of other studies, which have reported that increases in surface roughness facilitated bacterial colonization, resulting in increased bacterial adhesion [40,41]. Bacterial behavior varies depending on the size and topographic properties of TNTs. Shi et al. [42] reported that the number of bacteria cultured on the relatively rough surface of TNTs was significantly lower than the number of bacteria cultured on smooth Ti surfaces. The roughened nanointerface properties of TiO_2_ cause a stress response in some bacteria, leading to the rupture of the bacterial cell membrane and apoptosis. In addition, the adhesion of *S. mutans* onto rGO-deposited surfaces was further reduced compared with adhesion on the control group and TNT surfaces. The known antibacterial mechanisms of GO are as follows: (1) physical direct interaction of extremely sharp edges of nanomaterials with cell wall membrane [43], (2) ROS generation [44], (3) trapping the bacteria within the aggregated nanomaterials [45], (4) oxidative stress [46], (5) interruption in the glycolysis process of the cells, (6) DNA damaging [47], (7) metal ion release [48], and (8) contribution in generation/explosion of nanobubbles [49]. Bacterial adhesion was further reduced on the rGO-deposited surfaces after TNT formation, even more so than on the rGO-deposited surfaces alone. After the surface roughness increased with TNT formation, the bacterial cell wall membrane was directly damaged by the sharp edges of the deposited rGO, which appears to have further enhanced the antibacterial effect. In this study, it was found that rGO deposition decreased hydrophilicity and increased hydrophobicity. It has been reported that bacteria adhere better to hydrophobic surfaces than to hydrophilic ones [50]. The bacterium *S. mutans* used in this study is reportedly hydrophobic [51]. It can be expected that the surface rGO coating reduces hydrophilicity, thereby increasing the adhesion of the hydrophobic *S. mutans*. However, in this study, when comparing the control group and the rGO-deposited groups (Groups G and NG), it was observed that bacterial adhesion was significantly reduced in the rGO-deposited groups. Oxidative stress caused by rGO functional groups may damage biofilms on the specimens. Although the rGO coating reduced surface hydrophilicity and increased surface hydrophobicity, bacterial adhesion was reduced, as the increased hydrophobicity was offset by the physical properties of rGO.

Raman spectroscopy is widely used to characterize crystal structure, disorder, and defects in graphene-based materials [52]. In this study, we analyzed the samples using Raman spectroscopy to confirm the presence of rGO. Figure 5 shows the Raman spectra of rGO. The peak at 1350 and 1348 cm^−1^ in Group G and Group NG, respectively, corresponds to the D band, caused by first-order Raman scattering due to the E_2g_ mode following the Raman selection rule among Γ modes. This band is present in all sp^2^ carbon systems forming a C-C bond. The peak at 1593 and 1583 cm^−1^ in Group G and Group NG, respectively, corresponds to the G band, caused by the A_1g_ vibration mode, and its intensity can increase due to disorder structure of the plane. The peak at 2683 cm^−1^ in both Group G and Group NG represents the 2D band, indicating secondary scattering in which two phonons of D mode are emitted [53]. Figure 5 shows that the I_D_/I_G_ ratio of rGO shows an increasing trend in Group G (1.1) compared with in Group NG (1.3). This suggest that more graphitic domains are formed and the sp^2^ cluster number is increased [54]. Additionally, the number of graphene layers can be estimated through the I2D/G ratio. A 2D/G ratio of >2, 1~2, and <1 corresponds to single-layered, double-layered, and multi-layered graphene, respectively [53]. Therefore, it is evident that both Group G and Group NG are deposited with multi-layers of rGO, attributed to the synthesis method using nonthermal atmospheric plasma.

As for the changes in osteoblast activity, the adhesion and proliferation of MC3T3-E1 cells increased significantly in all three experimental groups compared with the control group. TNT formation on titanium surfaces also exhibits a higher proliferation rate than the control group. Oh et al. [55] reported that TNT arrays on titanium induced osteoblast proliferation by 300–400% compared to unmodified titanium surfaces. The reason for this is that the lateral spacing of nanoscale features can influence and change cell behavior [56]. The improved surface roughness of current bioactive implants is one of the important factors that provide appropriate clues to a good cellular response to the implanted material. Many studies on the effects of macro- and micro-roughness on cellular response and tissue formation have been inconclusive. However, it is reported that the increased roughness and surface area on the nanotube surface compared to unmodified titanium surfaces can influence and change certain cell behavior [14,57]. TNTs are known to have a greater surface energy than the untreated Ti [58]. It is reported that the contact angle, which means the wettability of the surface, is improved to be more hydrophilic on the nanotube surface, which is advantageous for improving protein adsorption and cell adhesion [59]. However, it has been reported that in the case of bacteria, TNTs can significantly reduce the number of cells attached to the surface [60]. Currently, opinions are inconsistent on how the hydrophilic/free properties of materials regulate bacterial cell behavior. Previous studies have reported that improving the hydrophilicity of the surface can promote bacterial attachment and proliferation, and that bacteria grow as the diameter of TNT increases [61,62]. However, Xiaoguo et al. [42] reported that as the diameter of TNTs increased, *P.gingivalis* tended to decrease and then increase again, indicating that there are complex factors controlling bacterial behavior at the biocompatible interface.

Item et al. [63] formed TNT on the surface of Ti6Al4V-ELI, electrophoretically deposited rGO, and reported the investigation of the antibacterial activity of *Staphylococcus aureus* and biocompatibility of L-929 fibroblast cells. TNT-rGO enhanced antibacterial activity without causing any morphological damage to bacteria, and for L-929 fibroblasts, rGO had a positive effect on cell adhesion and proliferation. In particular, the increase within the rGO-deposited groups was higher and statistically significant. The rGO formed in this study was deposited in the form of a cloud. Although it has a different shape from the surface utilized by Item et al. [63], the reason for the same results is thought to be because rGO has properties that promote the adhesion, proliferation, and differentiation of osteoblasts [58,59,60].

However, this study has some limitations in that all experiments were conducted in vitro. Although the roughness and surface area of TNTs can influence and change the behavior of bacteria and cells, this study tested only one size of TNT and rGO surfaces deposited under one condition. It is necessary to investigate TNT and rGO surfaces formed under various conditions and to evaluate changes in the properties of these surfaces and their activity against bacteria and cells. The results could differ if it was conducted in an oral environment with saliva. Also, only a single strain of *S. mutans* was used in this study, but the properties of bacteria itself can also influence the result [64]. The cell wall composition of Gram-negative bacteria is different from that of *S. mutans*, which indicates the different surface properties of bacteria. Hence, further studies are needed considering these limitations.

## 4. Materials and Methods

### 4.1. Samples

Pure titanium (ASTM Grade IV, Kobe Steel, Kobe, Japan) was prepared as disks with a thickness of 3 mm and a diameter of 15 mm. With a polishing machine (Labopol-5, Struers, Copenhagen, Denmark), the surface of each specimen was polished with 600-grit SiC abrasive paper under running water and sequentially up to 2000-grit SiC abrasive paper to finish. After the specimens were polished, they were ultrasonically cleaned using acetone, ethanol, and distilled water for 15 min each and dried. All specimens were sterilized with ethylene oxide (EO) gas.

### 4.2. Surface Treatment

#### 4.2.1. Anodic Oxidation

Anodic oxidation was performed using a DC power supply (Fine Power F-3005, SG EMD, Anyang, Republic of Korea). The electrolyte solution was prepared by adding 1 M phosphoric acid and 1.5 wt% hydrofluoric acid to distilled water. A platinum plate was connected to the cathode, and a titanium specimen was connected to the anode. The specimen and the platinum plate were dipped in the electrolyte solution, after which a voltage of 20 V was applied for ten minutes.

#### 4.2.2. Reduced Graphene Oxide Deposition

**Figure 10:**
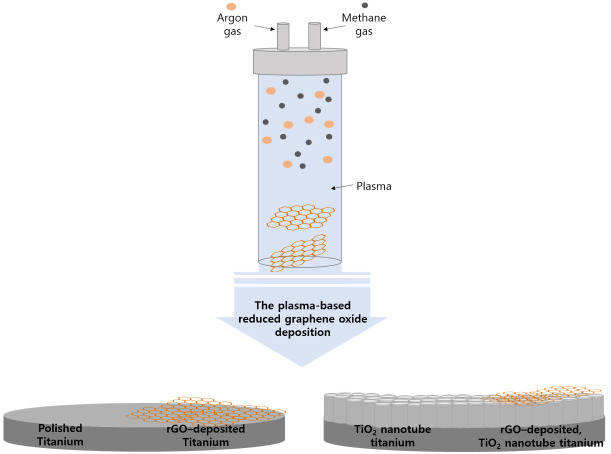
Schematic diagram of the atmospheric plasma-based reduced graphene oxide deposition.

Deposition of rGO onto the specimens was performed using a nonthermal atmospheric plasma generator (PGS-300, Expantech Co., Suwon, Republic of Korea). Argon gas (4 L/min) and methane gas (3.5 L/min) were mixed in a quartz tube to generate plasma, and this plasma was then applied to the specimens at a rate of 10 L/min with a power of 300 W using a plasma generator with a high-frequency (900 MHz) resonator (Table 1). The distance between the plasma flame and the specimens was maintained at 15 mm, and the plasma flame was moved from left to right while the specimens were rotated at 180 rpm so that the rGO could be evenly deposited. Plasma was applied for a total of six minutes per specimen by setting the plasma to reciprocate 24 times at 15 s per reciprocation. Figure 10 shows a schematic diagram of the atmospheric plasma-based rGO deposition.

### 4.3. Classification of Experimental Groups

The specimens used in this study were divided into a control group (polished titanium) and three experimental groups: Group N (TiO_2_ nanotube titanium), Group G (rGO-deposited titanium), and Group NG (rGO-deposited TiO_2_ nanotube titanium) (Table 2).

### 4.4. Assessment of Surface Characteristics

The surface structures of TNTs and rGO formed on the titanium specimens were observed using a field emission scanning electron microscope (S-4700, Hitachi, Horiba, Osaka, Japan). The surface roughness of the control and three experimental groups (N, G, and NG) was measured using a 2D contact stylus profilometer (DIAVITE DH-7, Asmeto AG, Schwyz, Switzerland). Surface roughness was measured at three points on each specimen, and the average of these three values was used as the specimen’s average roughness (Ra). To compare changes in the surface hydrophilicity of the specimens, 4 μL of distilled water was dropped on each specimen’s surface. The angle between the surface and the solution was measured after ten seconds using a video contact angle measuring device (Phoenix 300, SEO Co., Suwon, Republic of Korea). For each group, the contact angle of the specimens was measured and averaged. X-ray photoemission spectroscopy (XPS; MultiLab 2000, Thermo Electron Corporation, Warwickshire, UK) was performed to assess the elemental changes of the surfaces after rGO deposition. The area values of each peak for the detected elements were normalized and expressed as a quantitative ratio. Laser Raman spectroscopy (NRS-5100, JASCO, Tokyo, Japan) was performed to determine the rGO on the surface at 532.13 nm.

### 4.5. Assessment of the Ability to Inhibit Biofilm Formation

The ability of the surfaces to inhibit biofilm formation was assessed using a Gram-positive, facultative anaerobic bacterium, *S. mutans* (KCOM 1504), which causes early biofilm formation. *S. mutans* strains were purchased from the Korean Collection for Oral Microbiology (KCOM, Gwangju, Republic of Korea) and cultured in brain heart infusion (BHI; Becton, Dickinson and Company, Sparks, MD, USA) medium. A single colony of *S. mutans* formed on a solid medium was transferred into a liquid medium and cultured in an incubator (LIB-150M, DAIHAN Labtech Co., Namyangju, Republic of Korea) at 37 °C. Biofilm formation was assessed via a crystal violet staining assay. Bacteria at a concentration of 1.5 × 10^7^ CFU/mL were inoculated on the specimens, which were then cultured. After *S. mutans* was cultured for 24 h, the specimens were carefully washed twice with phosphate-buffered saline (PBS) to remove bacteria that had failed to attach to the specimens. A 0.3% crystal violet solution was dispensed onto the specimens, staining them for ten minutes. After removing the crystal violet solution using suction, the specimens were washed three times with PBS and dried for 15 min. A destaining solution (80% ethyl alcohol and 20% acetone) was dispensed onto the dried specimens and stirred for one hour. Then, 200 μL of the destaining solution was placed in a 96-well plate, and the absorbance was measured at 595 nm using a VersaMax ELISA microplate reader (Molecular Devices, San Jose, CA, USA).

### 4.6. Osteoblastic Activity

MC3T3-E1 mouse (*Mus musculus*) preosteoblastic cells were purchased from the American Type Culture Collection (ATCC; Manassas, VA, USA). The cells were cultured in an α-Minimum Essential Medium (α-MEM; Gibco-BRL, Grand Island, NY, USA) containing 10% fetal bovine serum (FBS) and 100 U/mL penicillin at 37 °C in a 5% CO_2_ culture incubator (FormaSeries II 3111 Water Jacketed CO_2_ Incubator, Thermo Scientific, Waltham, MA, USA). Culture media were replaced every three days, and the cells were subcultured until the number of cells was sufficient for the necessary tests. Four to seven generations of cells were used in this study. Ten specimens per group were placed in a 24-well plate, and 4 × 10^4^ cells/mL of MC3T3-E1 cells were dispensed onto the specimens and cultured in an incubator set at 5% CO_2_ and 37 °C for 24 and 72 h. After both incubation periods, to assess cell viability and proliferation, the WST-8 reagent (EZ-Cytox, Itsbio, Inc., Seoul, Republic of Korea) was dispensed into each well, and the plate was put into an incubator set at 37 °C and 5% CO_2_ for the reaction. When the orange color was developed by the WST-8 reagent, 100 μL of the culture medium containing the reagent was transferred from each well into a 96-well plate, and the absorbance was measured at 450 nm using a VersaMax ELISA microplate reader (Molecular Devices, San Jose, CA, USA).

The adhesion of MC3T3-E1 cells was observed using a scanning electron microscope (SEM). Two specimens per group were placed in a 24-well plate, and 4 × 10^4^ cells/mL of MC3T3-E1 cells were dispensed onto the specimens and cultured in an incubator set at 5% CO_2_ and 37 °C for 4 h. After 4 h of cell culture, the cells on the specimens were fixed in 2.5% glutaraldehyde for 2 h. After carefully washing with PBS solution twice, the cells were dehydrated in an ethanol gradient in the order of 40%, 50%, 60%, 70%, 80%, 90%, and 100% for 10 min at each concentration. The sample was dried on a clean bench for 2 h after the dehydration process. The cell morphology was observed using an SEM.

### 4.7. Statistical Analysis

For the statistical analyses conducted in this study, SPSS Statistics V21.0 (SPSS Inc., Chicago, IL, USA) was used. Data collected to evaluate biofilm-inhibition ability met the assumption of normality according to a Shapiro–Wilk test. Since the assumption of homogeneity of variance was also not violated, statistical analyses were performed using a parametric ANOVA and a post hoc Tukey test. The significance of all the results was tested at the level of a *p*-value less than 0.05. Since osteoblast adhesion and proliferation data did not satisfy the assumption of normality in the Shapiro–Wilk test, these data were statistically analyzed using a Kruskal–Wallis test, a nonparametric ANOVA method. After a Mann–Whitney *U* test was performed, the type I error was corrected using Bonferroni’s method to find groups of different sizes so that the significance was tested at a *p*-value less than 0.0083.

## 5. Conclusions

TNT formation and rGO deposition on a pure titanium surface decreased the adhesion of *S. mutans* at an early stage of 24 h (*p* < 0.05) and increased the adhesion and proliferation of MC3T3-E1 cells (*p* < 0.0083). The rGO-deposited surface with TNTs showed the lowest adhesion rate of *S. mutans* (*p* < 0.05) and the best proliferation of MC3T3-E1 cells (*p* < 0.0083).

Within the limits of this study, we suggest that TNT formation and rGO deposition on pure titanium surfaces can reduce the adhesion of *S. mutans* at an early stage. This surface modification is expected to improve the proliferation of preosteoblastic cells.

## Figures and Tables

**Table 1:** Parameters of the atmospheric plasma generator.

Parameter	Value
Average working power (W)	300
Voltage (V)	27
Frequency (MHz)	900
Atmospheric pressure (Torr)	760
Plasma density (cm^3^)	10^15^

**Table 2:** Experimental groups in this study.

Group	Coating Condition
Control	Polished titanium
N	TiO_2_ nanotube titanium
G	Reduced graphene oxide-deposited titanium
NG	Reduced graphene oxide-deposited TiO_2_ nanotube titanium

## Data Availability

Data are contained within the article.
